# Comment on: “Tumour-agnostic drugs in paediatric cancers”, Chisholm et al., *BJC* 2020

**DOI:** 10.1038/s41416-020-01103-0

**Published:** 2020-10-01

**Authors:** Thomas Richard William Oliver, Thomas John Jackson, Olumide Ogunbiyi, Neil Sebire, Olga Slater, Mette Jorgensen, Sam Behjati

**Affiliations:** 1 Wellcome Sanger Institute, Hinxton, CB10 1SA UK; 2 Cambridge University Hospitals NHS Foundation Trust, Cambridge, CB2 0QQ UK; 3 Great Ormond Street Hospital for Children NHS Foundation Trust, London, WC1N 3JH UK; 4 UCL Great Ormond Street Institute of Child Health, London, WC1N 1EH UK; 5 Department of Paediatrics, University of Cambridge, Cambridge, CB2 0QQ UK

**Keywords:** Molecular medicine, Sarcoma, Paediatric cancer

We agree in principle with our colleagues’ endorsement in this journal of the use of the tropomyosin receptor kinase (TRK) inhibitor LOXO-101 (larotrectinib) in children.^1^ Our enthusiasm for the accelerated deployment of LOXO-101 has been tempered by our experience of multi-TRK inhibitor resistance though. Resistance mutations to LOXO-101 do occur but can be overcome by second- generation TRK inhibitors, such as LOXO-195 (selitrectinib).^2^ We, however, report the emergence of resistance mutations to both first- and second-generation TRK inhibitors in an NTRK-driven infantile fibrosarcoma (IFS). Our case questions the introduction of TRK inhibitors into clinical practice without the scrutiny of comparative clinical trials, especially in the context of NTRK-driven childhood tumours that are rarely lethal. Our cautionary words are particularly pertinent in view of the recent approval to use LOXO-101 by the UK’s National Institute for Health and Care Excellence.

Our patient, a 10-month-old girl, suffered from an anterior mediastinal IFS extending into the neck that harboured a canonical IFS-associated *ETV6-NTRK3* gene fusion. We treated her over a period of 12.5 months with cytotoxic chemotherapy, first- and second-generation TRK inhibitors and two attempts at complete surgical resection (Fig. 1a, b). The tumour repeatedly regrew and ultimately led to the child’s death. To understand the development of treatment resistance, we reconstructed the evolution of the lethal clone from somatic mutations, obtained by whole genome (fatal relapse) or exome sequences (seven tissues obtained at diagnosis and after each recurrence). At diagnosis, the tumour contained single copies of wild-type *NTRK3* and the *ETV6-NTRK3* fusion (Fig. 1b). Analysis of the first relapse revealed duplication of the *ETV6-NTRK3* fusion. At second relapse, after 2 months of treatment with a first-generation TRK inhibitor (LOXO-101), we detected the *NTRK3* resistance mutation, G623R,^3^ along with loss of the wild-type *NTRK3* gene. Then, following 2.5 months of treatment with a second-generation TRK inhibitor (LOXO-195), multidrug resistance emerged in the shape of two mutations, *NTRK3* G623R and F617L.^3^ Both were present in the same cells of the dominant clone, as they were captured on the same reads (i.e. the same DNA molecule) (Fig. 1b). Reconstructing the phylogeny of tumour lineages from substitutions and small indels revealed a complex clonal composition of the tumour, from which the lethal, double- mutant clone emerged (Fig. 1c). These analyses delineate the development of a seemingly “supercharged” *ETV6-NTRK3* resistance fusion, forged under the selective pressure of TRK inhibitors.

**Fig. 1:**
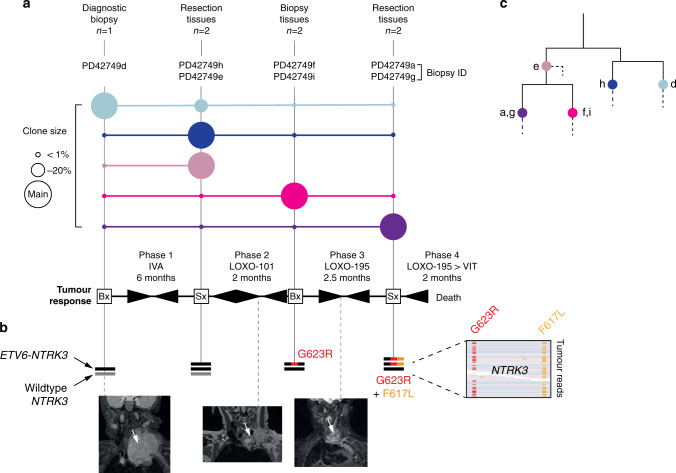
Evolution of multi-TRK resistance. **a** Clonal composition of tumour tissues over the course of illness. Coloured circles denote the clonal composition of tissues sequenced at a given time point. The scale of a clone’s contribution to these samples is relative to a circle’s size; larger circles indicate a greater contribution by that clone. Beneath this is a simplified overview of principal systemic therapies and the resultant tumour responses. IVA ifosfamide, vincristine, actinomycin D, VIT vincristine, irinotecan, temozolomide, LOXO-101 (larotrectinib) first-generation TRK inhibitor, LOXO-195 (selitrectinib) second-generation TRK inhibitor. Arrowheads tapering from left to right indicate partial/complete tumour response and vice versa for progression. **b** Sequence of somatic alterations of *NTRK3*. Black line: *ETV6-NTRK3* fusion. Grey line: wild-type copy of *NTRK3*. Number of lines corresponds to copy number. Screen capture on the right shows the co-occurrence of both resistance mutations on the same tumour sequencing reads. At the bottom are three coronal MRI images of the tumour (white arrow): one at diagnosis (left) and two demonstrating the tumour’s best response to LOXO-101 (middle) and LOXO-195 (right). **c** Phylogenetic tree of tumour lineages. Solid lines: major branches where mapped mutations revealed the relationship of the clones identified. Labelled overlying coloured circles represent the clones shown in panel **a**. Dashed lines: private mutations that did not inform tree building.

The key evidence justifying the use of LOXO-101 in children with NTRK-driven tumours is a phase 1/2 study, which showed an impressive 93% objective response rate (ORR) amongst 15 children.^4^ Two of these with locally advanced disease even achieved R0 resections following treatment with TRK inhibitors. We note that one patient did develop the same *NTRK3* G623R resistance mutation as our patient although responded to a second-generation inhibitor (selitrectinib). Recent work in a mixed-age cohort across histologies suggests that a 45% ORR can be achieved with LOXO-195 in patients with these NTRK kinase domain mutations.^3^

On this basis, it would appear that TRK inhibition is an attractive, non-cytotoxic strategy for tumour shrinkage prior to, or even in lieu of, surgery in childhood tumours of low metastatic potential, such as IFS. Our challenge has been that the same marked clinical responses to both TRK inhibitors were seen in our patient, yet were short lived and preceded the outgrowth of a lethal tumour clone. Without access to long-term efficacy data or head-to-head comparisons to best current practice, it is difficult to know how representative our experience is and so highlights the current challenge in identifying the most opportune moment to utilise TRK inhibitors in the management of IFS. Clearly, in the context of invariably lethal cancers, phase 1/2 studies may provide sufficient evidence for utilising tumour-agnostic drugs. However, in seldom lethal NTRK-driven childhood tumours, we require comparative clinical trials beyond basket studies to learn when and how to deploy these drugs. Until such evidence is available, we would advocate a cautious use of TRK inhibitors in children.

## Data Availability

Raw sequencing data pertaining to this case are available in EGA.
